# Visual assessment of pancreatic fat deposition: useful grading system and the relation to BMI and diabetes

**DOI:** 10.1007/s11604-022-01334-6

**Published:** 2022-09-13

**Authors:** Ryusuke Ookura, Noriaki Usuki

**Affiliations:** Department of Diagnostic and Interventional Radiology, Japan Community Healthcare Organization Osaka Hospital, 4-2-78, Fukushima, Fukushima-ku, Osaka, 553-0003 Japan

**Keywords:** Pancreatic disease, Non-alcoholic fatty pancreas disease, Pancreatic steatosis, Obesity, Diabetes mellitus

## Abstract

**Purpose:**

To establish a simple and clinically useful method for the visual assessment of pancreatic fat deposition using computed tomography (CT) images, and to evaluate the relationship of the pancreatic fat deposition with body mass index (BMI) and type 2 diabetes mellitus (DM).

**Materials and methods:**

We used a four-scale grading system as the visual assessment criteria for pancreatic fat deposition using CT images. Pancreatic fat deposition was assessed for 200 patients and the results were compared with the CT attenuation-based assessment. In addition, the relationships of pancreatic fat deposition with BMI and type 2 DM were investigated.

**Results:**

The visual and CT attenuation-based assessments were considered consistent. The results of the visual assessment suggested that mild and moderate pancreatic fat deposition correlated with BMI and presence of type 2 DM while severe fat deposition did not correlate with them. No correlation between pancreatic fat deposition and HbA1c level was found.

**Conclusion:**

The visual assessment criteria we used were consistent with CT attenuation-based assessment and may be useful for clinical application of pancreatic fat deposition. According to the visually assessment, mild or moderate pancreatic fat deposition correlated with BMI and the presence of type 2 DM, but severe fat deposition did not correlate with them.

## Introduction

In abdominal imaging, pancreatic fat deposition is a common finding. It has been suggested that pancreatic fat deposition is a risk factor for metabolic syndrome [1, 2], non-alcoholic fatty liver disease (NAFLD) [3], and type 2 diabetes mellitus (DM) [4, 5]. The term “non-alcoholic fatty pancreas disease (NAFPD)” or “fatty pancreas” has become common due to its importance. Also, it has recently been suggested that pancreatic fat deposition is associated with acute pancreatitis [6, 7], pancreatic cancer [8–10] and postoperative pancreatic fistula [11–14]. Although the clinical significance of pancreatic fat deposition is still unknown, it is expected to become more important to evaluate it easily and accurately in the future.

Histopathological evaluation is the most accurate method to assess pancreatic fat deposition, however, it is highly invasive for clinical use. Imaging studies such as ultrasound (US), computed tomography (CT), and magnetic resonance imaging (MRI) are usually performed for the assessment of pancreatic fat deposition. In the assessment of pancreatic fat deposition using CT, it is common to estimate the fat content by assigning regions of interest (ROI) at several locations in the pancreas and measuring the CT values. However, this method is complicated, and a simpler method for evaluating pancreatic fat deposition is needed for clinical application. In addition, the assessment of fat deposition by CT value can be applied only to plain CT and not applicable to contrast-enhanced CT or MRI.

In this study, we used a grading system for the visual assessment criteria for pancreatic fat deposition and examined whether there was any consistency with the CT attenuation-based assessment. In addition, we investigated the relationship of the visually assessed pancreatic fat deposition with body mass index (BMI) and presence of type 2 DM.

## Materials and methods

### Grading system for visual assessment of the pancreatic fat deposition

In this study, we used the following grading system which is an improved assessment criteria for the pancreatic fat deposition using CT by Marks et al. [15]. Briefly, we combined the four grades of Marks’ criteria into three grades (0–2) and separated advanced fat deposition, in which more than half of the pancreas is replaced by fat, as grade 3.

Grade 0:Normal. Pancreatic parenchyma is homogeneous, with smooth or mildly lobulated margins and no fat tissue is observed in the pancreatic parenchyma.

Grade 1:Mild. The margin of the pancreas is serrated but the deep pancreatic parenchyma (around main pancreatic duct) is homogeneous. Mildly low-attenuated areas in the pancreatic parenchyma may be seen, but no coarse fat tissue is observed.

Grade 2:Moderate. Coarse fat tissues are scattered and extends to the deep pancreatic parenchyma.

Grade 3:Severe. More than half of the pancreas shows isoattenuation with fat.

**Fig. 1:**
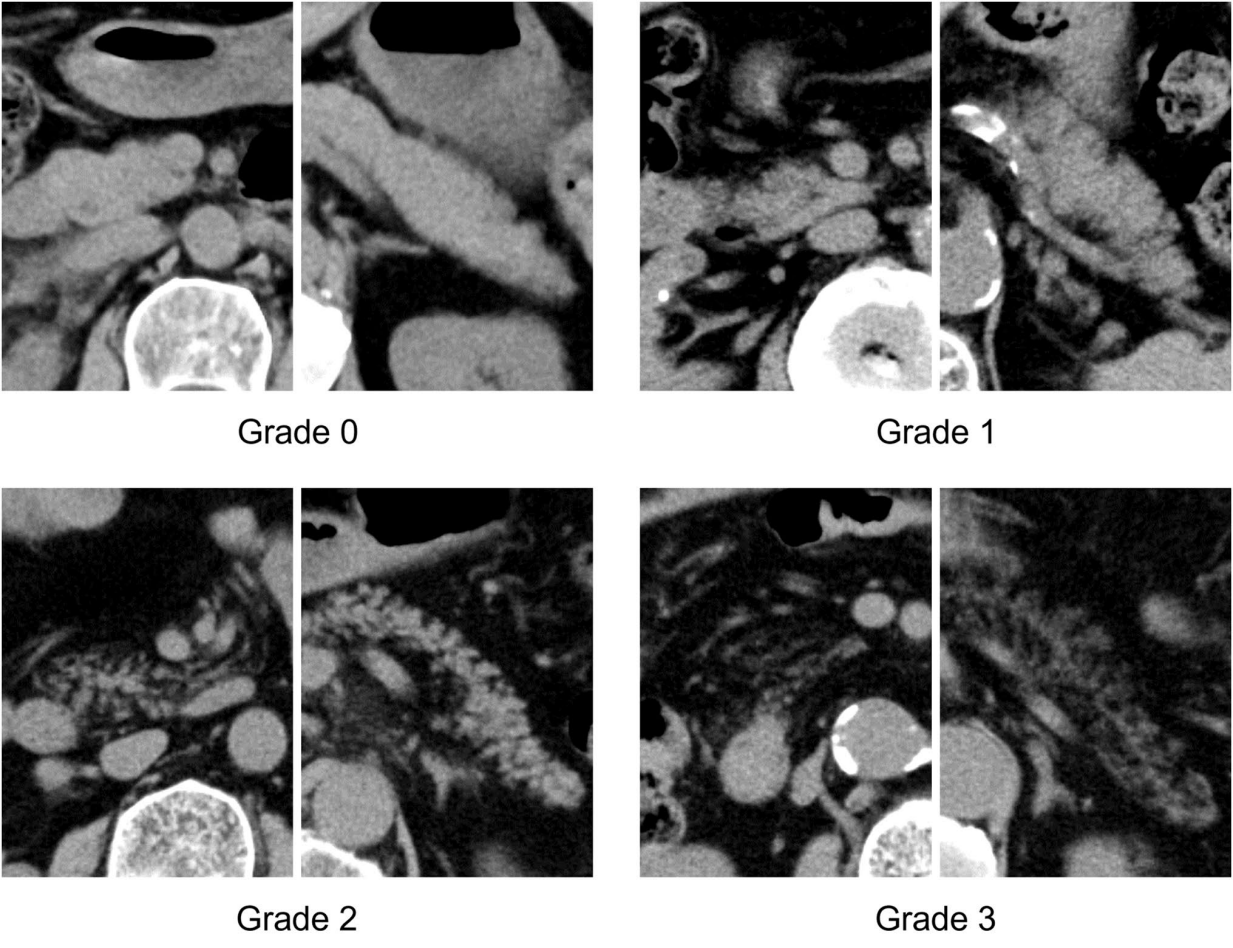
Representative images of each grade of the visual assessment. Left: pancreas head, right: pancreas tail

Typical CT images of each grade are shown in Fig. 1.

### Study participants

The ethics committee of our hospital approved the use of image data and the retrospective study design. This study was carried out according to the Declaration of Helsinki.

From the patients who underwent plain CT of the upper abdomen at our hospital between April 2021 and September 2021, we selected the patients who were classified to be grade 0–3 by the above visual assessment criteria, respectively. Patients under the age of 20 years, within a month after abdominal surgery, indwelling tube in bile duct or pancreatic duct, acute abdomen for any causes, diagnosed as pancreas diseases (e.g., acute pancreatitis, chronic pancreatitis, intraductal papillary mucinous neoplasm (IPMN), pancreatic cancer), and cases in which pancreatic morphological assessment was difficult due to choledocholithiasis, large periampullary diverticulum and severe artifacts were not included. If several CT scans were performed on the same patient within the study period, the easiest one to assess was selected.

We also calculated BMI from the height and weight entered in the information of Digital Imaging and Communications in Medicine (DICOM) at the time of CT acquisition. Cases in which either height or weight was not input in the DICOM information were not included.

### Visual assessment of the pancreatic fat deposition

For all the patients, visual assessment was performed at two locations, the head and tail of the pancreas. For the pancreatic head, the portion between the Vater papilla and uncinate process was selected. For the pancreatic tail, the middle portion between the intersection of the pancreas and the left border of the aorta and the lateral end of the pancreas was selected. In cases where it was difficult to decide between the two grades, the lower one was adopted.

### Imaging acquisition and CT attenuation-based assessment

**Fig. 2:**
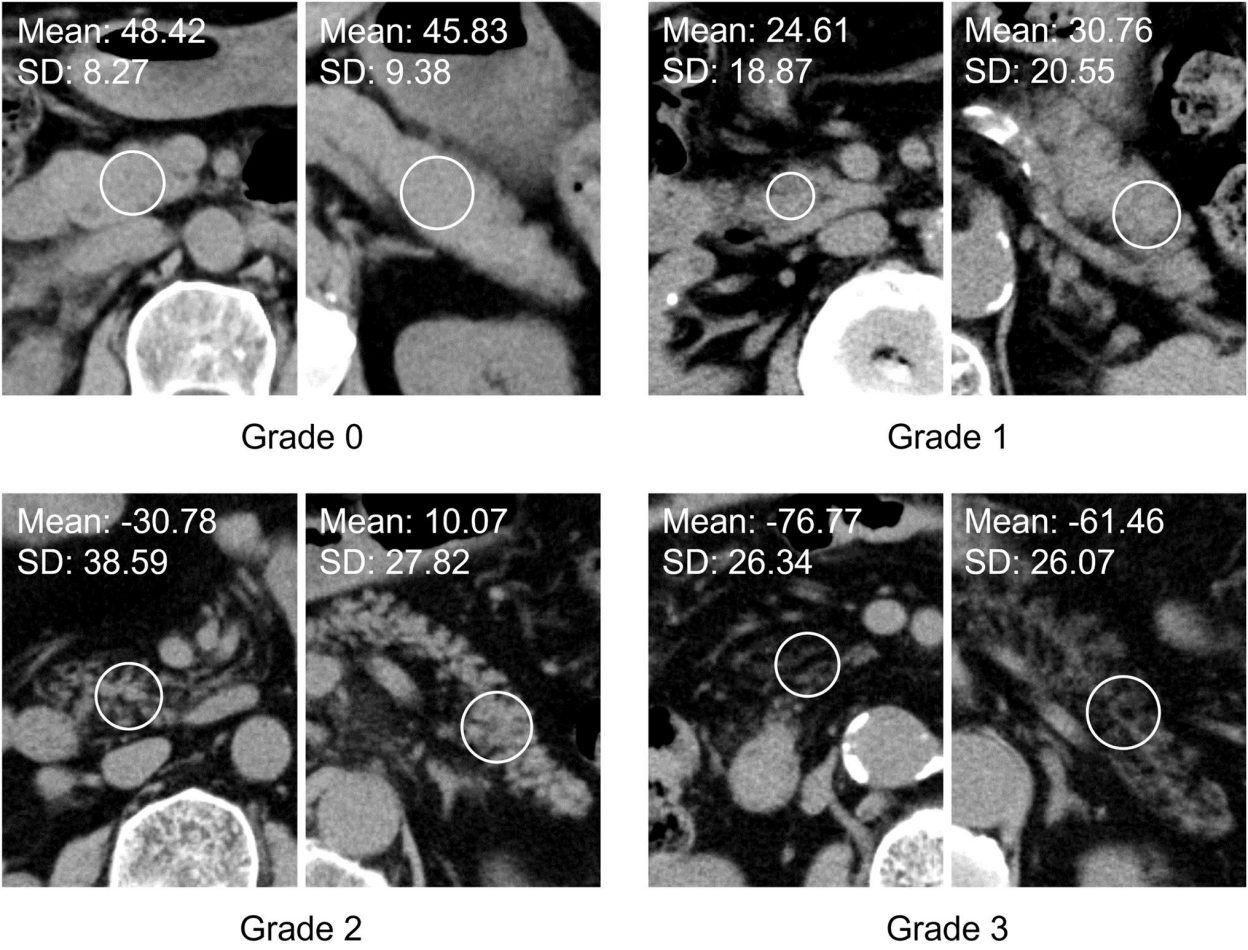
The method of ROI assignment for the CT attenuation-based assessment on pancreas head (left) and tail (right) on the same images of Fig. 1. Mean and standard deviation of CT values of each ROI are shown

Abdominal CT was performed using 64- or 80-detector-row scanners (Aquilion, Toshiba Medical Systems, Tokyo, Japan). Images were acquired with 0.5–0.75 s rotation time, 1.0-(64-detector-row scanner) or 0.5-(80-detector-row scanner) mm beam collimation, pitch factor of 0.8, tube voltage of 120 kV, and 32 ~ 36 cm scanning field of view. Imaging analysis was performed on the 5 mm thickness reconstruction slices, because it is commonly used thickness clinically. Circular ROI were assigned at the pancreas head and tail on the CT images, and the mean and standard deviation (SD) of the CT value was measured. The measurement sites of the pancreas head and tail were set at the same position as the visual assessment. To reflect the fat deposition on the pancreatic margin, one ROI was assigned from anterior to posterior edge of the pancreas excluding common bile duct, portal vein and artifacts, and including main pancreatic duct and intrapancreatic fat tissue (Fig. 2).

To evaluate the intraobserver variability, the visual assessments were performed twice by one board-certified radiologist with an interval of more than one week. In addition, to evaluate the interobserver variability, another board-certified radiologist performed the assessment independently. Quadratic weighted kappa coefficients were calculated from the concordance rates of each assessment.

### The correlation between pancreatic fat deposition and BMI

We investigated the relationship between the grade of pancreatic fat deposition and BMI. Three results of the visual assessment were examined: the first and the second visual assessment of one radiologist for intraobserver variability evaluation, and the visual assessment of the other radiologist for the interobserver variability evaluation.

In addition, the correlations between BMI and CT values of the pancreatic head and tail were examined using Spearman’s rank correlation coefficient (*R*).

### The correlation between pancreatic fat deposition and type 2 DM

We investigated the relationship between the grade of pancreatic fat deposition and the presence of type 2 DM. For each case, the presence or absence of type 2 DM and the HbA1c level within 1 month before and after CT acquisition were extracted from the medical records. If several HbA1c levels were available on the same patient, the closest one to CT acquisition was selected. Cases diagnosed as type 1 diabetes were excluded. We investigated the correlation of the grades of fat deposition with the presence of type 2 DM and HbA1c level.

In addition, the correlations between the presence of DM and CT values of the pancreatic head and tail were examined using Student’s *t* test, and the correlations between HbA1c level and the CT values were examined using Spearman’s *R*.

### Statistical analysis

Statistical analysis was performed using EZR software (version 1.50) [16]. Statistical comparisons were made by Kruskal–Wallis test followed by Steel–Dwass post hoc test for the ratio or interval scales variables and by Fisher’s exact test with Bonferroni correction for the ordinary or nominal scales. Values of *p *< 0.05 was considered statistically significant.

## Result

### Patients

A total of 200 patients were included. The mean age was 63.5 years (20–96 years), and the gender was 96 males and 104 females. The patient profile by grade is shown in Table 1.

**Table 1:** Overview of patient profile

	*N*	Age (years, mean ± SD)	Gender (male: female)
Pancreas head			
Grade 0	112	58.6 ± 20.8	41:71
Grade 1	32	68.0 ± 17.5	23:9
Grade 2	34	66.4 ± 14.0	21:13
Grade 3	22	77.0 ± 10.9	11:11
Pancreas tail			
Grade 0	117	58.4 ± 20.9	44:73
Grade 1	47	67.7 ± 14.6	31:16
Grade 2	25	71.7 ± 12.9	15:10
Grade 3	11	80.6 ± 7.9	6:5

There were significant differences in patient age between grade 0 and grade 3 (*p* < 0.001), grade 2 and grade 3 (*p* = 0.0253) for the pancreatic head, and between grade 0 and grade 3 (*p* = 0.00428) and grade 1 and grade 3 (*p* = 0.0401) for the pancreatic tail, respectively.

### The consistency between visual and CT attenuation-based assessment

**Fig. 3:**
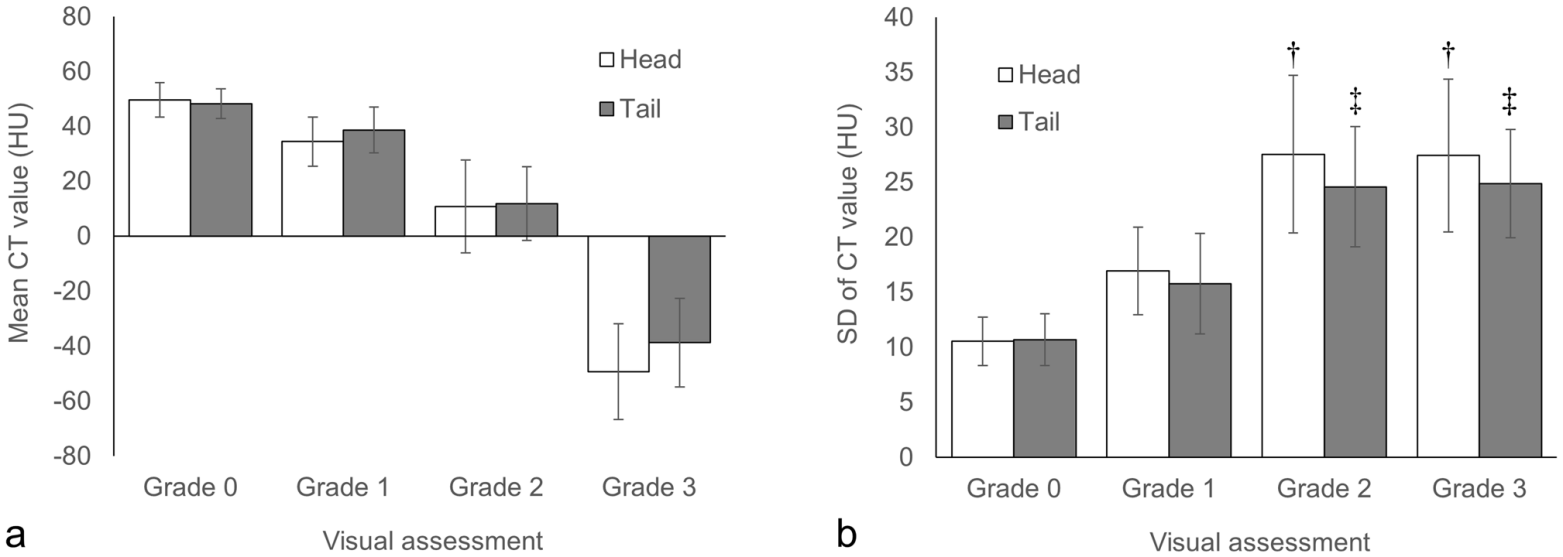
Relationship between visual and CT attenuation-based assessment. Mean (**a**) and SD (**b**) of CT values in ROI. *No significant difference was found* only between the SD of grade 2 and 3 in both the head (†) and tail (‡) of the pancreas (both *p* = 1.00), however, *p* < 0.00005 for all other combinations. For the rest of them, there were significant differences between all grades in both the head and tail

The relationship between the visual and CT attenuation-based assessment is shown in Fig. 3. The mean CT values (Hounsfield unit (HU), mean ± SD) of each grade are as following; pancreatic head, grade 0: 49.7 ± 6.3HU, grade 1: 34.4 ± 9.0HU, grade 2: 10.7 ± 16.9HU, grade 3: − 49.3 ± 17.4HU; pancreatic tail, grade 0: 48.3 ± 5.5HU, grade 1: 38.6 ± 8.4HU, grade 2: 11.8 ± 13.5HU, grade 3: − 38.7 ± 16.1HU. The SD of CT values in ROI (mean ± SD) of each grade are as following: Pancreatic head, grade 0: 10.5 ± 2.2HU, grade 1: 16.9 ± 4.0HU, grade 2: 27.5 ± 7.2HU, grade 3: 27.4 ± 7.0HU. Pancreatic tail, grade 0: 10.7 ± 2.4HU, grade 1: 15.8 ± 4.6HU, grade 2: 24.6 ± 5.5HU, grade 3: 24.9 ± 4.9HU.

The concordance rate between the two measurements by one radiologist was 84.5% (169/200) for the pancreatic head and 87.0% (174/200) for the pancreatic tail, and the weighted kappa coefficient was 0.925 (95% confidence interval (CI 0.888–0.963) for the pancreatic head and 0.921 (CI 0.883–0.960) for the pancreatic tail. The concordance rate of the measurements by the two radiologists was 67.5% (135/200) for the pancreatic head and 60.5% (121/200) for the pancreatic tail, and the weighted kappa coefficient was 0.830 (CI 0.774–0.886) for the pancreatic head and 0.698 (CI 0.624–0.772) for the pancreatic tail. Because the kappa coefficients were enough high, both intraobserver and interobserver variability were considered to be sufficiently small.

### Correlation between visual assessment of pancreatic fat deposition and BMI

**Fig. 4:**
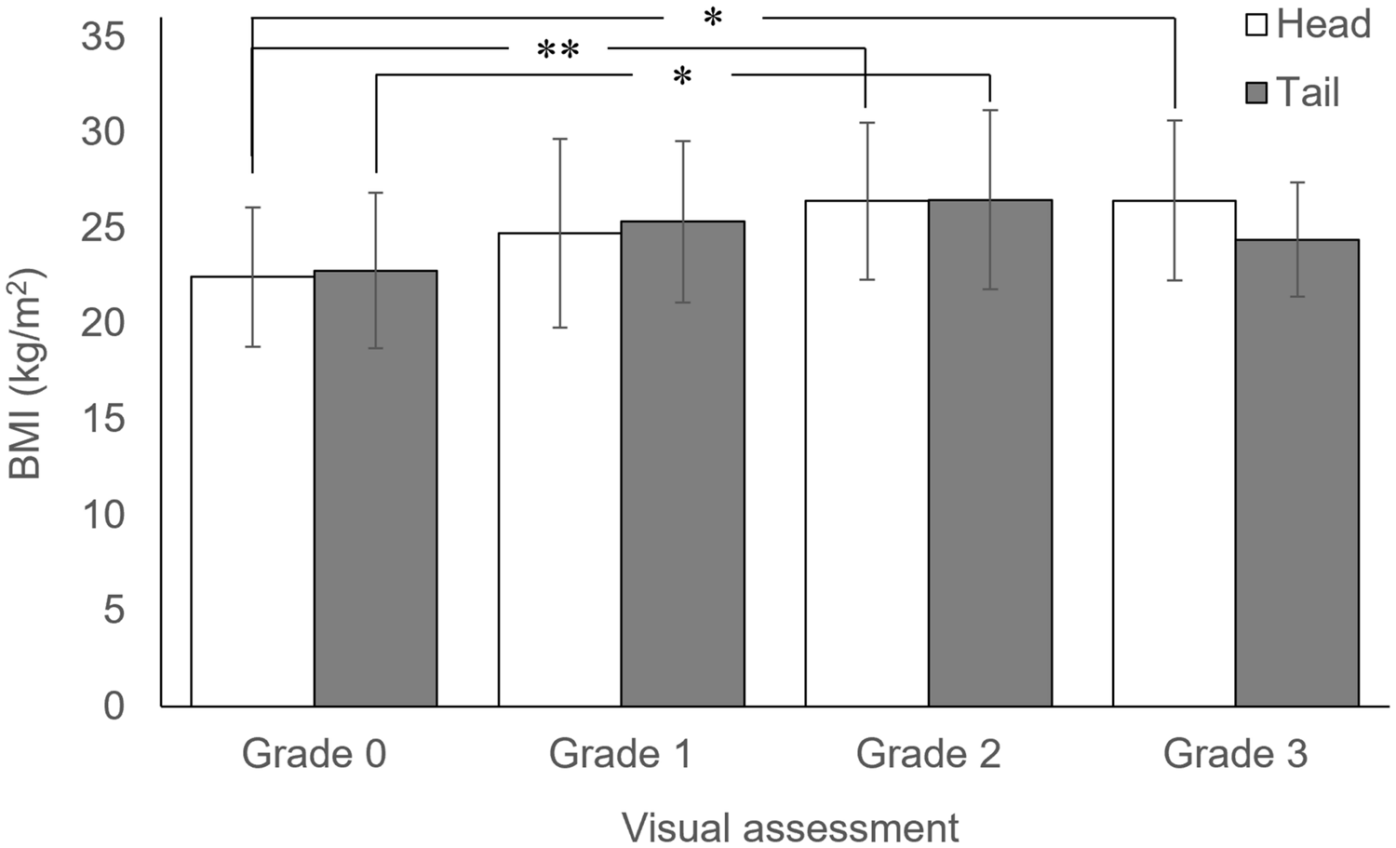
Relationship between visual assessment and BMI. The graph shows the mean and SD of the results of first visual assessment. *: *p* < 0.005 and **: *p* < 0.00001, respectively for all three results of visual assessment consistently. In both the head and tail of the pancreas, no significant difference was observed between the patients with glade 1 and the higher

The relationship between the grade of pancreatic fat deposition and BMI is shown in Fig. 4. The BMI (kg/m^2^, mean ± SD) of each grade are as following; Pancreatic head, grade 0: 22.4 ± 3.7, grade 1: 24.7 ± 4.9, grade 2: 26.4 ± 4.1, grade 3: 26.4 ± 4.2; pancreatic tail, grade 0: 22.8 ± 4.1, grade 1: 25.3 ± 4.2, grade 2: 26.5 ± 4.7, grade 3: 24.4 ± 3.0.

Using all three results of the visual assessment, we examined the correlation between the grade and BMI. The following results were obtained consistently. For the head of the pancreas, BMI was significantly higher in grades 2 (*p* < 0.001) and 3 (*p *< 0.001) compared to grade 0. For the tail of the pancreas, BMI was significantly higher in grade 2 compared to grade 0 (*p* = 0.00160). There was no significant difference in BMI between grades 1 and 3 and between grades 2 and 3 in both the head and tail of the pancreas.

Spearman’s *R* between BMI and CT values were − 0.431 (*p* < 0.001) for the pancreatic head and − 0.362 (*p* < 0.001) for the pancreatic tail.

### The correlation between pancreatic fat deposition and presence of type 2 DM

**Fig. 5:**
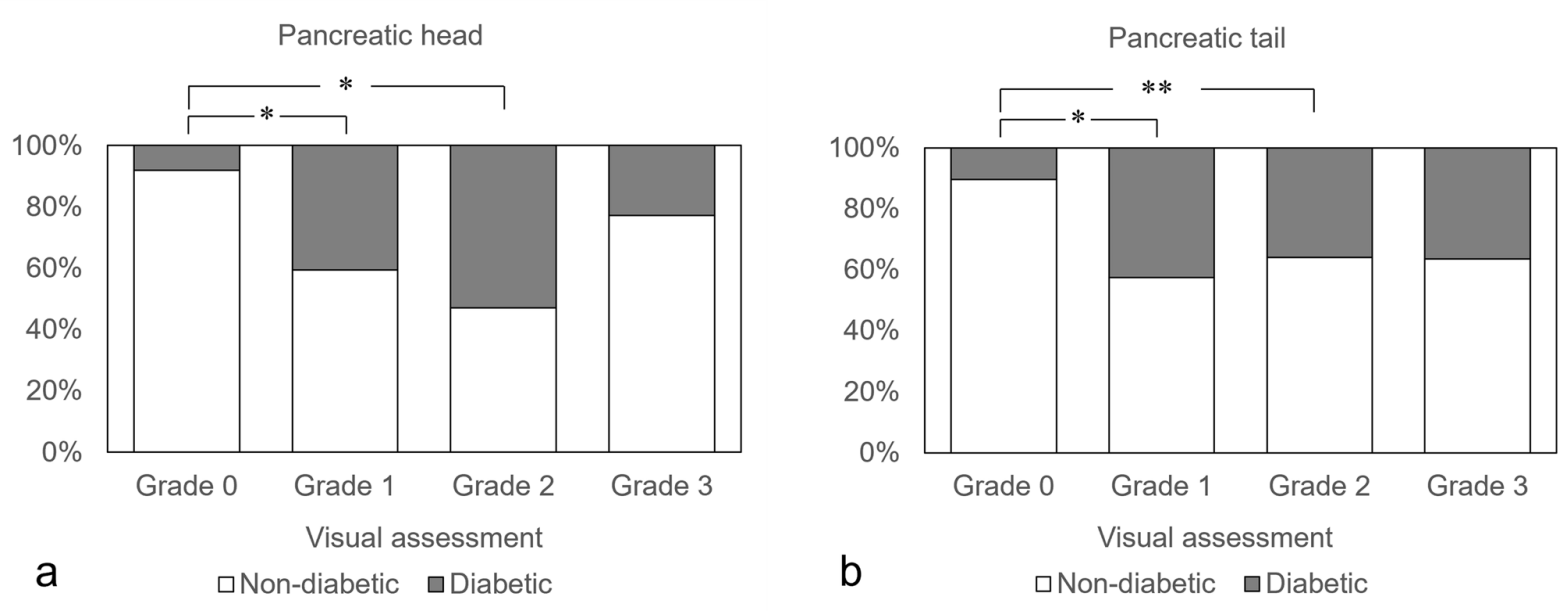
The ratio of the cases with and without type 2 DM in each grade of pancreatic fat deposition. *: *p* < 0.0001 and **: *p* < 0.05, respectively

The relationship between the grade of pancreatic fat deposition and presence of type 2 DM is shown in Fig. 5. According to the medical record, 45 patients were diagnosed with type 2 DM. One patient diagnosed as type 1 DM was excluded. The prevalence of type 2 DM was significantly higher in grades 1 and 2 than in grade 0 in both the head and tail (head; grade 1: *p* < 0.001, grade 2: *p* < 0.001, tail; grade 1: *p *< 0.001, grade 2: *p* = 0.0180).

The relationship between the CT attenuation-based assessment for pancreatic fat deposition and presence of type 2 DM is shown in Table 2. The significant correlations of the presence of DM and CT value were found both the head and tail of the pancreas.

**Table 2:** Correlation between the presence of DM and CT value

	CT value (HU, mean ± SD)		*p* (Student’s *t*-test)
	Non-diabetic	Diabetic	
Pancreas head	33.2 ± 32.7	17.2 ± 31.6	0.00401
Pancreas tail	39.1 ± 22.1	28.0 ± 25.4	0.00494

*DM* diabetes mellitus, *HU* Hounsfield unit

HbA1c level was measured in 60 cases during the study period. The HbA1c level (%, mean ± SD) of each grade are as following; pancreatic head, grade 0: 6.1 ± 0.8, grade 1: 6.4 ± 1.3, grade 2: 6.7 ± 0.9, grade 3: 6.8 ± 2.4; pancreatic tail, grade 0: 6.0 ± 0.7, grade 1: 6.7 ± 1.2, grade 2: 6.3 ± 0.8, grade 3: 8.1 ± 3.3. No significant differences were observed between any grades both the head and tail of the pancreas. Spearman’s *R* between HbA1c and CT value were − 0.255 (*p* = 0.049) for the pancreatic head and − 0.201 (*p* = 0.123) for the pancreatic tail. These results showed that there was no significant correlation between the HbA1c level and pancreatic fat deposition both the head and tail of the pancreas.

## Discussion

Since the study of Marks et al. [15], there have been many studies using visual assessment of pancreatic fat deposition by US and CT [17]. After that, there are some studies on the visual assessment of pancreatic fat deposition using US, however, there are few studies on the visual assessment using CT or MRI. Recently, Hori et al. reported that CT-area-based assessment was more accurate than CT-attenuation-based assessment for the evaluation of pancreatic fat deposition by CT [18]. The visual assessment used in this study can be positioned as a more simplified and clinically accessible version of such CT-area based assessment. Using such criteria, clinicians can estimate the degree of pancreatic fat deposition at a glance and may become easily to estimate the presence of metabolic syndrome and DM or to predict the risk of complications after pancreatic surgery.

In this study, as the grade of visual assessment increased, the mean CT value decreased, and the SD of CT value increased. Therefore, pancreatic fat deposition was presumed to progress according to the grade of the visual assessment. Pancreatic fat deposition can be divided into three categories according to its histological location: intralobular, interlobular, and extralobular/peripancreatic [19]. In our visual assessment, it is considered to be equivalent to the grade 0 for normal, grade 1 for intralobular to interlobular, grade 2 for interlobular, and grade 3 for interlobular to extralobular/peripancreatic. Therefore, it is presumed that histological pancreatic fat deposition also generally progresses in this order.

In many previous studies using CT, ROIs are placed in each of the head, body and tail of the pancreas and the average of CT values is measured. However, the degree of fat deposition in the pancreas is not homogeneous and it is more severe in the pancreas head (especially in the anterior aspect) than body and tail [20]. This suggests that the causes and clinical significance of fat deposition may differ between the head and tail of the pancreas. Considering this, we assessed the pancreatic head and tail separately. The results showed that the head is more prone to fat deposition than the tail, which is consistent with previous studies.

Kim et al. proposed a threshold of < 36 HU for the diagnosis of pancreatic steatosis [21]. Although the method of measuring CT values is different, this threshold may correspond to glade 1 in our visual assessment. Fine adipose tissue within the pancreas or serrated changes in the pancreatic margins are likely to be the morphologic changes of the pancreatic fat deposition that are initially detectable on imaging studies.

In this study, only plain CT was used, because CT attenuation-based assessment was used for comparison with the visual assessment. However, because only morphology is assessed in the visual assessment, it is also applicable to contrast-enhanced CT and MRI and these may be rather more accurate than plain CT.

Regarding the relationship between pancreatic fat deposition and BMI, patients with grade 2 or 3 of the pancreatic head had higher BMI than those with grade 0, and those with grade 2 of the pancreatic tail had higher BMI than those with grade 0. On the other hand, no significant difference of BMI was observed between the patients with glade 1 and the higher grades in both the head and tail of the pancreas. This suggests that obesity is likely to be related with the development of mild and moderate pancreatic fat deposition, but not related with the severe fat deposition. It is assumed that other factors may play a significant role in the severe pancreatic fat deposition. Only weak correlation was observed between BMI and CT values in both the head and tail from Speareman’s correlation coefficient, probably because the correlation is partial and not linear.

Regarding the relationship between pancreatic fat deposition and DM, the CT value of the pancreas in the cases with type 2 DM was significantly lower than in the cases without type 2 DM in both the head and tail. On visual assessment, the prevalence rate of type 2 DM was higher in the grade 1 and 2 cases than in the grade 0 in both the head and tail. These results show that cases with pancreatic fat deposition are more likely to have type 2 DM in the background, which is consistent with previous reports [4, 5]. However, there was no significant difference between grade 3 and grade 0. From these results, it is inferred that grade 3 has different factors from grades 1 and 2, similar to the results seen in relation to BMI.

The correlation between the grades 0–2 fat deposition and BMI or the presence of DM was not seen in the grade 3. One possible reason for this is that grade 3 may be more affected by age and other factors than BMI and DM. In particular, grade 3 cases were significantly older than other grades. It is known that aging is also one of the causes of pancreatic fat deposition [22]. The results of this study suggest that the severe fat deposition, such as grade 3, is unlikely to be caused by BMI or DM alone and is presumably caused by the addition of age and other factors.

In this study, both on the CT attenuation-based assessment and on the visual assessment, pancreatic fat deposition correlated with the presence of DM, while it did not correlate with HbA1c. This may suggest that pancreatic fat deposition is associated with the presence of DM but not with their control nor severity. There are several possible hypotheses for this, for example (1) the pancreatic fat deposition affects to the onset of DM but not to the progression, or (2) pancreatic fat deposition due to obesity improves according to the DM progression.

There are some limitations of this study. First, our visual assessment was compared with the CT attenuation-based assessment, but not with the histopathological assessment. To do this assessment, surgical or autopsy cases with prior imaging studies are necessary. Future research is needed.

Second, pancreatic atrophy was not considered. Although it is known that the pancreas atrophies with age, we assessed the morphology ignoring the atrophy. It is unclear whether it is appropriate to assess the pancreas with or without atrophy by the same criteria.

Third, because this study relied on medical record for the diagnosis of DM, it is possible that some diabetic cases were included in the non-diabetic group. In addition, the impact of DM treatment on pancreatic fat deposition is not considered.

In conclusion, the grading system we used for visual assessment criteria were consistent with CT attenuation-based assessment and may be useful for clinical application of pancreatic fat deposition. When the presence of pancreatic fat deposition was found on the images, it may suggest the presence of obesity or type 2 DM. However, severe pancreatic fat deposition in which more than half of the pancreas is replaced by fat cannot necessarily be explained only by obesity or type 2 DM, suggesting that some other factor may be more significantly affect.
